# Exploratory Application of Augmented Reality/Mixed Reality Devices for Acute Care Procedure Training

**DOI:** 10.5811/westjem.2017.10.35026

**Published:** 2017-12-14

**Authors:** Leo Kobayashi, Xiao Chi Zhang, Scott A. Collins, Naz Karim, Derek L. Merck

**Affiliations:** * Alpert Medical School of Brown University, Department of Emergency Medicine, Providence, Rhode Island; † Rhode Island Hospital, CT Scan Department, Providence, Rhode Island; ‡ Alpert Medical School of Brown University, Department of Diagnostic Imaging, Providence, Rhode Island

## Abstract

**Introduction:**

Augmented reality (AR), mixed reality (MR), and virtual reality devices are enabling technologies that may facilitate effective communication in healthcare between those with information and knowledge (clinician/specialist; expert; educator) and those seeking understanding and insight (patient/family; non-expert; learner). Investigators initiated an exploratory program to enable the study of AR/MR use-cases in acute care clinical and instructional settings.

**Methods:**

Academic clinician educators, computer scientists, and diagnostic imaging specialists conducted a proof-of-concept project to 1) implement a core holoimaging pipeline infrastructure and open-access repository at the study institution, and 2) use novel AR/MR techniques on off-the-shelf devices with holoimages generated by the infrastructure to demonstrate their potential role in the instructive communication of complex medical information.

**Results:**

The study team successfully developed a medical holoimaging infrastructure methodology to identify, retrieve, and manipulate real patients’ de-identified computed tomography and magnetic resonance imagesets for rendering, packaging, transfer, and display of modular holoimages onto AR/MR headset devices and connected displays. Holoimages containing key segmentations of cervical and thoracic anatomic structures and pathology were overlaid and registered onto physical task trainers for simulation-based “blind insertion” invasive procedural training. During the session, learners experienced and used task-relevant anatomic holoimages for central venous catheter and tube thoracostomy insertion training with enhanced visual cues and haptic feedback. Direct instructor access into the learner’s AR/MR headset view of the task trainer was achieved for visual-axis interactive instructional guidance.

**Conclusion:**

Investigators implemented a core holoimaging pipeline infrastructure and modular open-access repository to generate and enable access to modular holoimages during exploratory pilot stage applications for invasive procedure training that featured innovative AR/MR techniques on off-the-shelf headset devices.

## INTRODUCTION

Technologic advances have enabled commercially available virtual reality (VR) devices such as the HTC Vive and Oculus Rift to immerse end-users in convincing, artificial environments that (re-)create dramatic and engaging perceptual experiences through visual, auditory, and haptic signals. However, these worlds are accessible only when users wear opaque VR goggles and relinquish several essential and interactive aspects of the physical realm of reality. In contrast, augmented reality (AR) overlays a supplemental digital realm *onto* the real world through various devices (*e.g.,* visors, smartphones), enabling users to continue to interact with their physical surroundings while simultaneously experiencing and interacting with digital objects and artifacts linked to actual environmental elements. (The associated concept of mixed reality [MR] encompasses all combinations of real, augmented, and virtual environments.^1^) A recreational yet acutely illustrative example is that of Pokémon Go (Niantic, San Francisco, CA), a popular mobile AR game in which players see and interact with digitally rendered creatures in video-captured, real-world landscapes on their AR device screens.^2^

Beyond consumer-focused uses such as gaming and social media, AR/MR technologies harbor dramatic potential for meaningful scientific application due to their ability to radically shift the way individuals interact with other people, places, objects, and ideas. Specifically, the representation and sharing of informative data through sophisticated visualization approaches is likely to blossom into an explosive phenomenon. AR/MR is already catalyzing healthcare and medical education, as it embodies an innovative and accessible approach for clinicians, patients, researchers, and educators to see, discuss, study, experiment, implement, and share complex concepts. Established examples in diverse fields include anatomy education,^3^–^5^ general healthcare,^6^–^9^ and procedural preparation or training for acute care medicine,^10^–^14^ dentistry,^15^ general surgery^16^–^20^ neurosurgery,^21^,^22^ ophthalmology,^23^ orthopedics,^24^,^25^ urology,^26^ and vascular surgery.^27^ Given this background, a team of academic clinician educators, computer scientists, and diagnostic imaging specialists conducted a proof-of-concept project to apply AR/MR to specialized acute care procedure training.

## OBJECTIVES

Our goals were as follows: 1) Implement a basic holoimaging pipeline infrastructure with open-access repository to facilitate exploratory applications of AR/MR at the study institution and beyond; and 2) Use novel AR/MR techniques on off-the-shelf devices with holoimages generated by the infrastructure to demonstrate their potential role in the instructive communication of complex medical information.

## METHODS

### Curricular Design and Implementation for Exploratory Application

Program investigators structured an exploratory research framework to examine AR/MR’s unique capabilities, implementation needs and barriers, and use characteristics in accessible, high-yield, best-case healthcare settings (Appendices 1a–1b). For one of the program’s initial focus points for AR/MR clinical application, investigators proposed the use of AR/MR-enhanced instructional guidance to train emergency medicine (EM) learners in central venous line (CVL) placement and tube thoracostomy insertions, i.e., specific, common, invasive, and important acute care therapeutic interventions that feature consistent internal anatomic structures. As both procedures are performed with ultrasound guidance as the current standard of care, the training of acute care providers in “blind” CVL and tube thoracostomy insertions was targeted as a potential exemplar application of instructional AR/MR visualization in light of the potential for ultrasound device malfunction (*e.g.,* probe failure) and non-availability.^28^–^30^ Updating and expanding on historic, landmark-based training that predated widespread ultrasound use, investigators planned to use AR/MR devices experimentally to combine 3D internal anatomic-model visualizations with interactive task training that featured haptic feedback and kinesthetic learning in real time at the educational bedside.

To set up the envisioned training application, the team accessed two department-funded, off-the-shelf HoloLens headsets ($3,000 per unit; Microsoft, Redmond WA) at a hospital simulation center. Specifically, these devices were used to expand an existing hands-on procedural teaching curriculum in place for EM fellows, residents, advanced practice providers, and medical students. We designed a two-hour pilot session to accommodate 40 learners for the “blind insertion” procedural training using two distinct and complementary approaches: 1) modular AR/MR holoimaging overlay and registration of patient anatomic structures and pathology onto procedural task trainers; and 2) first-person view visual-axis interactive instructional guidance. The institutional review board approved the project protocol.

### Core Medical Holoimaging Pipeline Infrastructure and Open-Access Modular Holoimage Repository

Diagnostic imaging and computer science co-investigators collaborated with clinician educators to develop and assemble a medical holoimaging pipeline infrastructure with the following elements:

1. Query mechanism to identify, locate, and retrieve radiographic imagesets with specified clinical findings in the hospital network’s Centricity Picture Archiving and Communication Systems (PACS; GE, Chicago IL) using mPower (Nuance, Burlington MA)
2. Imageset manipulation approach for de-identified, thin-slice, contrast-enhanced trauma “pan-scan” computed tomography (CT) volumetric imagesets to create modular and discrete regional segmentations of anatomic structures and pathologic findings of interest:
  2a. We extracted structures of interest with voxel intensity-based growing algorithms using an Advantage Workstation (GE, Chicago IL).
  2b. We converted 3D scalar fields of structures of interest into polygonal mesh isosurfaces as an .stl file (3D Systems, Valencia CA) with a marching cubes algorithm, using iNtuition (TeraRecon, Foster City CA).
  2c. We managed mesh smoothness and vertex counts using open-source MeshLab (ISTI-CNR, Italy) to fit within the processing capabilities of select AR/MR headset devices.
3. Compositing, rendering, and packaging process with differentiation of mesh model layers by color and synthesis of integrating holoimages using Maya software for export in .fbx format (Autodesk, San Rafael CA)
4. Transfer mechanism to move holoimages onto institution-approved secure network storage, cloud storage solution, and/or an open-access repository
5. Retrieval and display procedure to access holoimages on AR/MR headset devices.

In its current implementation, the process pipeline required approximately 30 minutes for a typical head or chest CT imageset from the time of patient selection until holoimage display (the feasibility of partially automating holoimage processing is being studied). One specific advantage of developing this type of infrastructure was the ever-expanding repository of modular holoimages that can be subsequently retrieved and displayed, either as originally rendered or in assembled *holocomposites* that reflect patterns of pathology, e.g.*,* combinations of multi-organ traumatic injury holoimages. At an administrative level, procedures and protocols for formalization of the infrastructure and dissemination mechanisms for institution-wide use are under development in parallel with discussions of staffing and funding arrangements. In the interim, approximately 20 of the developed holoimage .fbx files are accessibly stored on a storage cloud repository located at: https://repository.library.brown.edu/studio/collections/id_753/ (open access and download functionality).

### Registered Overlaid Holoimaging for Anatomic Visualization

**Figure 1:**
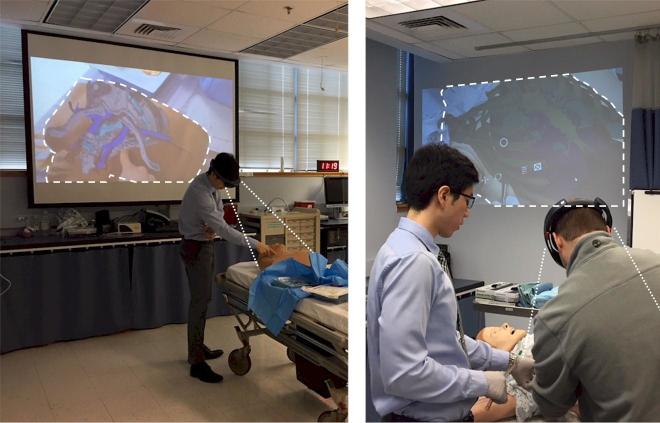
Left image: Wireless broadcasting of augmented reality/mixed reality (AR/MR) headset first-person-view videostream for shared anatomic visualization during central venous line training. Right image: AR/MR-enhanced tube thoracostomy training with highlighted outline of real-time projected visual overlay of pneumothorax and thoracic structures.

Overlaying real patients’ holoimages of key cervical and thoracic structures onto physical task trainers was essential to provide learners with the equivalent of a “visible human” for the explicit purpose of conveying the locations, shapes, sizes, and juxtapositions of internal vascular and thoracic anatomic structures with respect to surface features. This was accomplished by displaying the rendered holoimages on the HoloLens units with 3DViewer Beta (Microsoft) during the practice insertion of invasive therapeutic devices (Figure 1). The holoimages were manually scaled and registered onto the CVL task trainer (Blue Phantom/CAE Healthcare, Sarasota FL) and tube thoracostomy-compatible SimMan 3G manikin (Laerdal, Wappingers Falls NY) using surface anatomy landmarks, e.g*.,* sternal notch, laryngeal prominence, rib spaces. This permitted the stable overlay of task-relevant anatomy, e.g*.,* internal jugular, carotid artery; ribcage, lung parenchyma, and pneumothorax, for training with enhanced visual cues and haptic feedback. A wirelessly networked laptop connected with the AR/MR device’s built-in webportal system to broadcast the learner’s view with superimposed holoimages for the viewer cohort.

### Visual-Axis Interactive Instructional Guidance

**Figure 2:**
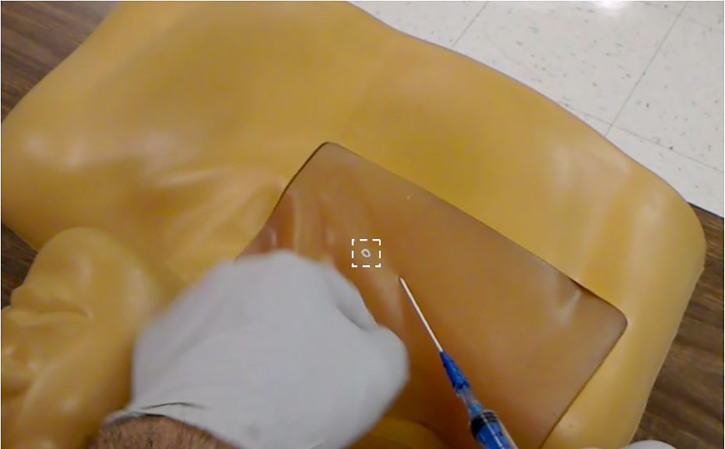
Still image of augmented reality/mixed reality (AR/MR) headset first-person view during visual-axis interactive instructional guidance for central venous line insertion training. The small pointer (highlighted by box outline) in the learner’s visual field is controlled by the instructor and serves as a dynamic marker to help the learner position and direct the catheter for an optimal vessel cannulation approach.

The second instructional approach exploited wireless AR/MR-enhanced Skype video-conferencing (Microsoft) between the learner’s HoloLens headset and an educator laptop with LCD projector. This arrangement was critical for accessing the learner’s AR/MR headset view of the task trainer for visual-axis interactive instructional guidance, i.e*.,* the introduction of educator-inserted 3D visual cues such as digital pointers and arrows. By using the headset’s Skype functionality, the learner video-called an educator’s laptop Skype application with HoloLens Add-in (with screen-mirroring projection of the learner FPV). This resulted in lagless sharing of the learner’s procedural performance perspective along with the ability for both the learner and educator to “draw” into the shared view using an on-screen toolset, e.g*.,* digital pencil tool or arrow tool. For environments without network connectivity or Skype, an alternative approach has been tested using an ad hoc device-laptop connection and Bluetooth-connected pointing device such as a wireless mouse to remotely direct the learner’s attention (Figure 2).

## RESULTS

### Proof-of-Concept Simulation Session

Investigators successfully applied the described AR/MR holoimage visualization methodologies to a two-hour pilot session with approximately 40 learners. Four 30-minute breakout sessions (with two educators each instructing groups of 10 participants) were completed as part of a scheduled EM residency conference period; simulation and standardized patient scenarios were conducted in parallel sessions during the AR/MR-enhanced invasive procedural training. To emphasize anatomic visualization with AR/MR assistance and to review “blind insertion” techniques, the learners were intentionally not provided with ultrasound devices; standard approaches, equipment, and procedural kits were otherwise used for both procedures. All participants finished both task-training exercises without physical discomfort; formal objective metrics were not obtained during this proof-of-concept session. See Appendix 2 for .mp4 video of AR/MR headset first-person view of a de-identified patient’s thoracic anatomy 3D model overlaid onto a patient simulator.

## DISCUSSION

Investigators successfully piloted the use of off-the-shelf AR/MR devices to integrate holoimaging-enhanced anatomic visualization with acute care invasive procedure training. We developed and implemented novel methodologies, including a core institutional holoimaging pipeline infrastructure, open-access storage repository of modular holoimage segmentations, and visual axis instruction techniques, for education and research purposes.

Program researchers are continuing to explore AR/MR applications in acute care, surgical care, and medical science while concurrently working to formally study the utility and usability of the developed AR/MR methodologies in healthcare. As part of the research program’s multiphasic strategy, investigations of AR/MR devices at the live clinical bedside are being prepared in peri-procedural settings: anatomic and diagnostic imaging AR/MR visualizations will be shown to patients and family members to gauge how the intervention facilitates discussions regarding management options and whether it assists with shared decision-making. Simultaneous, shared holoimage visualization across multiple headsets/viewers has already been accomplished at the study site; automation of holoimage registration with the real world for improved user experiences is being actively pursued.

With respect to future directions, the program team is pursuing ongoing use of AR/MR supplementation during EM residency sessions to objectively examine its educational utility for acute care procedural training. Defined metrics such as operational quality markers (e.g*.,* holoimage registration accuracy, stability, and usability) and longitudinal, checklist-based procedural performance assessments in simulated settings will need to be established and validated. Additional acute care procedural training that could uniquely benefit from AR/MR-enabled anatomic referencing and guidance are being reviewed, e.g*.,* arthrocentesis, epistaxis control, lumbar puncture, orthopedic manipulations, and complex wound exploration. Well-designed trials will be necessary to ascertain the effectiveness of AR/MR-based training and treatment interventions on live-patient clinical outcomes.

By sharing developed materials through open-access publications and online digital repositories, the research team is working to promote intramural and extramural efforts for widespread and collaborative efforts to investigate AR/MR techniques and technologies. Such efforts, the resulting use-case explorations, protocols, standards, and technical advancements (along with the expected release of lower-cost headset devices from several manufacturers) are anticipated to begin laying the foundation for meaningful and judicious use of AR/MR in healthcare.

### Limitations

The program’s core holoimaging infrastructure was made possible by the significant in-kind efforts of co-investigators (DLM, SAC) with deep, authorized access into the institution’s clinical diagnostic imaging systems. The primary operational limitation encountered by the research team derived from the manual overlay and registration process, in that the spatial alignment between the holoimage and the task trainer was intermittently lost and required adjustments. We did not assess or compare the effects of the experimental AR/MR approach on learner training and performance against existing methods during this initial pilot application. As a novel visualization approach that has only recently become more accessible to healthcare providers and researchers, holoimaging is anticipated to progress through the “hype cycle” framework,^31^ with initial enthusiasm, media highlighting, followed by unrealistic expectations, disillusionment, and then eventual adoption with evolution of the technology.

## CONCLUSION

Investigators have focused on the technical aspects of core system implementation, such that continued efforts will need to address the substantial work that remains in applying human factors engineering, ensuring institutional acceptance, and conducting empirical investigations into the clinical utility of holoimaging’. Previous work has already identified a variety of emergent AR-specific adverse effects in the clinical environment, e.g*.,* distortion of real-world perception,^32^ navigational and interface challenges,^33^ and inattentional blindness.^34^ Due diligence will be necessary to ensure its safe and effective application.

## Supplementary Information
