# Supplementary material for: Exploratory Application of Augmented Reality/Mixed Reality Devices for Acute Care Procedure Training
Source: West J Emerg Med. 2017 Dec 14;19(1):158–64. doi: 10.5811/westjem.2017.10.35026 (PMC5785186; doi:10.5811/westjem.2017.10.35026)
Supplement: Supplementary file 1 [file wjem-19-158-s001.pdf]

Appendix 1a: AR/MR exploration program modular framework with risk assessment and anticipated results. The table outlines the overall structure of the ongoing, collaborative, multi-disciplinary AR/MR exploration program as currently envisioned. The highlighted section indicates the acute care procedure training exploration elements discussed in the manuscript.

**Collaboration Focus and Field**	**Medical Information Communication**	**Surgical Anatomic Referencing**		**Translational Investigation Facilitation**		
	**Emergency Department**	**Neurosurgery**	**Plastic + Reconstructive Surgery**	**Diagnostic Imaging**	**Rehabilitation Medicine**	**Emergency Medicine Education/Training**
**Infrastructure Implementation Risks and Impact**						
1a. Risk assessment	*High risk*: AR/MR device linking, ED deployment pending; end-user buy-in unknown	*Considerable risk*: AR/MR device compatibility with OR suite and workflow unknown	*Considerable risk*: AR/MR device compatibility with OR suite and workflow unknown	*Moderate risk*: Holoimaging compatibility and utility for proposed uses unknown	*High risk*: Hardware + software interface development required	*Low risk*: Emergency Medicine PoC complete-> scope and scalability unknown
1b. Long-term potential impact if successful	Profound shift in provider-patient communication / information / SDM	Substantial advancement of operative planning and visualization	Substantial advancement of operative planning and visualization	Expansion of holoimaging approach to diverse scientific efforts	Significant impact on disability rehabilitation costs + resource access	Improved procedural success and safety with better training + online supervision
**Anticipated Data and Output for Reporting, Packaging, and Dissemination**						
2a. AR-enhanced intervention implementation	Shared visualization of neurologic emergencies (neoplastic, traumatic, vascular)	Semi-automated, pre-/intra-operative holoimaging display with alignment mechanism	Semi-automated, pre-/intra-operative holoimaging display with alignment mechanism	3 separate projects -*Neurology/ Neuroscience*: Retinal OCT visualization -*Evolutionary Biology*: Fossil model imaging -*Spine Surgery*: Spine screw placement 3D visualization	AR-enhanced system prototype(s) for gait and upper limb rehabilitation	AR-enhanced procedural training (endotracheal intubation; central line placement)
2a1. Exploration metrics	1-month followup SDM metrics (control and holo-image groups)	Patient-holoimage FPV alignment precision on video review	On-site (office) SDM metrics (control and holo-image groups)		Completion of functional prototype with assessment instruments	First-pass success<br>Time to placement<br>Number of attempts<br>Complications
2a2. Sample size	n=30+ subjects (LARs;15+ control, 15+ experimental)	n=~10 intra-operative uses	n=~10 outpatients (office) and ~10 intra-operative uses		n=1-2 pilot subjects	n=~40 subjects<br>-Simulation sessions (10 learners/session)<br>-Live environment: (~2 learners/shift)
2a3. Analysis methods and results	Non-parametric between-group comparisons	Descriptive and quantitative analyses of holo-image alignment	Descriptive and quantitative analyses of holo-imaging utility	Descriptive analyses of holo-imaging utility	Iterative prototype research + development	Non-parametric between-group comparisons
2b. Example next-step investigations identified for study	Expansion to ED holoimaging of thoracic/abdominal pathology	Development of automating software for patient-holoimage registration + stereo-tactic interfacing	Development of automating software for patient-holoimage registration	Continued application of shared visualization for new scientific efforts + insights	Formal study of prototype rehabil-itation systems with larger cohorts (n=10-15)	Formal integration of AR-enhanced procedural education into clinical rotations
**Anticipated Output for Reporting, Packaging, and Dissemination**						
3a. Baseline Assessment	Healthcare AR exploration knowledgebase, derived from review of literature and ongoing work, heuristic assessments, real-time provider observations, focus group interviews, contextual inquires, U-FMEA					
3b. Example HFE metrics	Emergency physician interviews, SUS, contextual inquiry<br>LAR followup SUS<br>AR/MR device operational characteristics (ED)	Surgeon interviews, SUS, contextual inquiry<br>AR/MR device operational characteristics (ED)	Surgeon interviews, SUS, contextual inquiry<br>Patient interviews, SUS<br>AR/MR device operational characteristics (ED)	Researcher interviews, SUS, contextual inquiry<br>AR/MR device development environment characteristics	Researcher interviews, SUS, contextual inquiry<br>AR/MR device development environment characteristics	Emergency Medicine educator interviews, SUS, contextual inquiry<br>Learner SUS, contextual inquiry<br>AR/MR device operational characteristics (simulation; ?live ED)
3c. HFE Package	Compiled findings from use-case exploration HFE datasets for AR/MR healthcare implementations with lessons learned and best practices as a shared resource					

Key: AR = augmented reality ED = Emergency Department FPV = first-person view HFE = human factors engineering LAR = legally authorized representative OCT = optical coherence tomography OR = operating room PoC = proof-of-concept SDM = shared decision making SUS = System Usability Scale U-FMEA = user failure modes effects analysis

Appendix 1b: Diagram of research holoimaging infrastructure to support AR/MR exploratory methodology and use-case applications. The diagram visually represents the currently established system and processes in place at the study institution for identifying, retrieving, manipulating, rendering, packaging, transferring, and displaying modular holo-images on AR/MR headset devices and connected displays. The highlighted section indicates the elements used during the acute care procedure training exploration discussed in the manuscript; ongoing AR/MR programs are accessing the remaining elements.

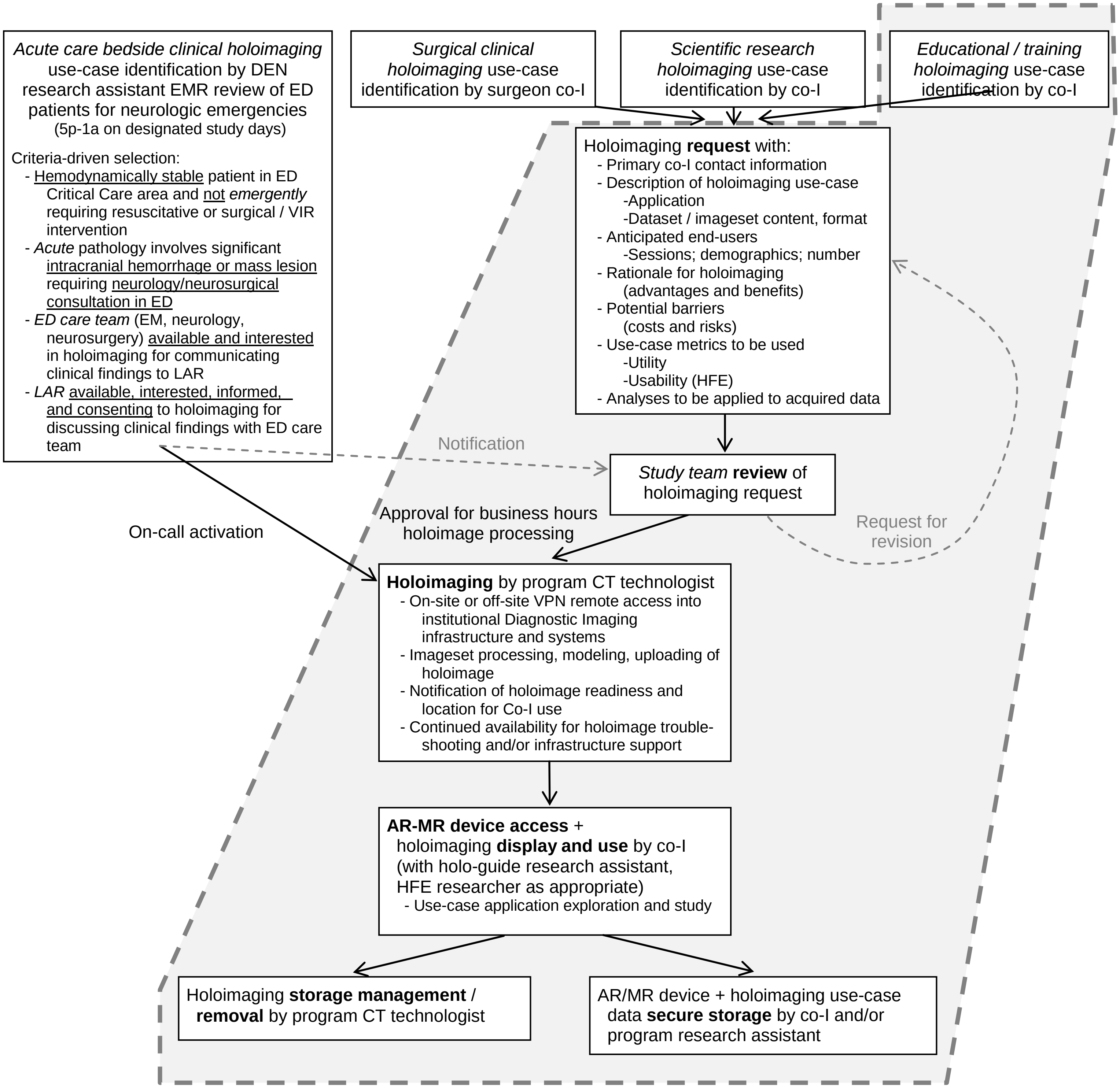

Key: co-I = co-investigator ED = Emergency Department DEN = Division of Emergency Neurosciences (RIH ED) EMR = electronic medical record HFE = human factors engineering LAR = legally authorized representative VIR = vascular interventional radiology VPN = virtual private network
