# Supplementary material for: Exploratory Application of Augmented Reality/Mixed Reality Devices for Acute Care Procedure Training
Source: West J Emerg Med. 2017 Dec 14;19(1):158–64. doi: 10.5811/westjem.2017.10.35026 (PMC5785186; doi:10.5811/westjem.2017.10.35026)

Appendix 2: First-person-view videostream of AR/MR anatomic model overlay onto simulator. .mp4 video of AR/MR headset first-person view of a de-identified patient's thoracic anatomy 3D model overlaid onto a patient simulator.

file: AR-MR Aim2 education PoC (anatomic pathology overlay) 9.24.2016
link: https://www.dropbox.com/s/wgta7unuspwxy0j/AR-MR%20Aim2%20education%20PoC%20%28anatomic%20pathology%20overlay%29%209.24.2016.mp4?dl=0

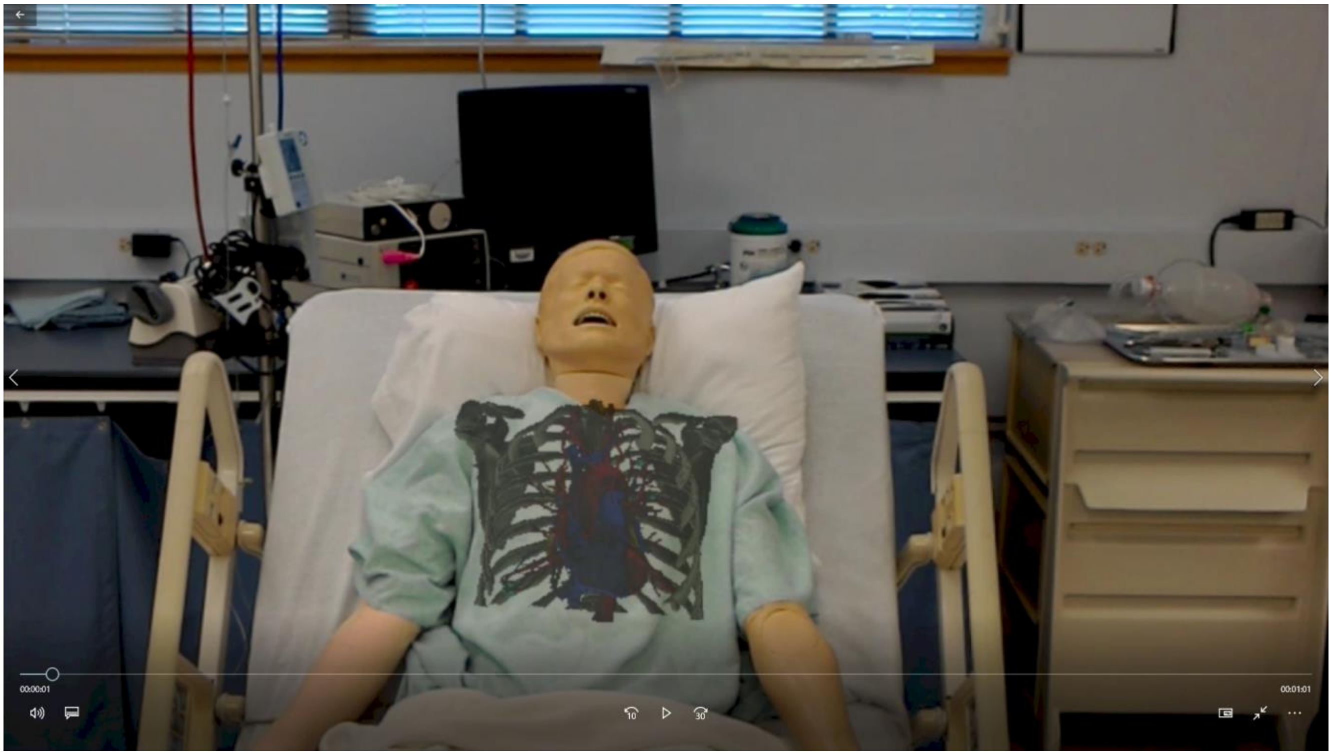

Supplement: Supplementary file 2 [file wjem-19-158-s002.pdf]
